# Supplementary material for: Gut Microbiome Dysbiosis in Patients with Pemphigus and Correlation with Pathogenic Autoantibodies
Source: Biomolecules. 2024 Jul 22;14(7):880. doi: 10.3390/biom14070880 (PMC11274803; doi:10.3390/biom14070880)
Supplement: Supplementary file 1 [file biomolecules-14-00880-s001.zip › Figure S.pdf]

Supplementary Materials

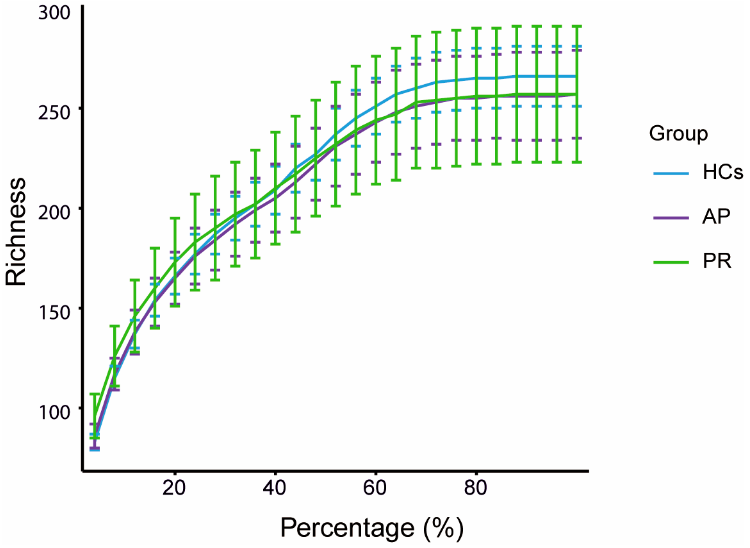

Figure S1: The rarefaction curve depicting species richness. AP group, active pemphigus group; PR group, pemphigus remission group; HCs group, healthy controls group.

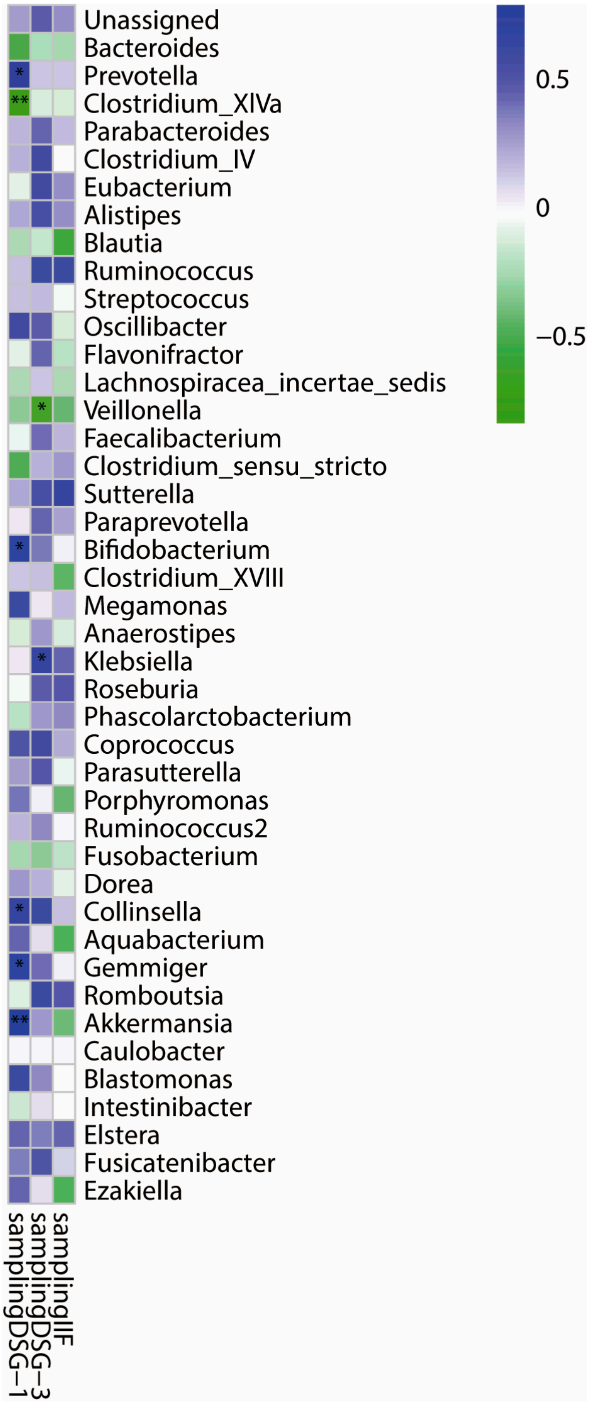

Figure S2: Spearman correlations between differential ASVs and clinical indicators, where $p < 0.01$ is denoted by ** and $p < 0.05$ by *.
