# Supplementary material for: Gut Microbiome Dysbiosis in Patients with Pemphigus and Correlation with Pathogenic Autoantibodies
Source: Biomolecules. 2024 Jul 22;14(7):880. doi: 10.3390/biom14070880 (PMC11274803; doi:10.3390/biom14070880)
Supplement: Supplementary file 1 [file biomolecules-14-00880-s001.zip › Table S.pdf]

Table S1 Differential ASVs depiction between the HCs and AP groups.

ID	P-Value	level	MeanHCs	MeanPP	Kingdom	Phylum	Class	Order	Family	Genus	Species
ASV_114	7.22E-07	Depleted	0.001	0.604	Bacteria	Proteobacteria	Alphaproteobacteria	Caulobacterales	Caulobacteraceae	Caulobacter	C.segnis
ASV_68	4.25E-06	Depleted	0.003	0.392	Bacteria	Firmicutes	Clostridia	Clostridiales	Ruminococcaceae	Faecalibacterium	F.prausnitzii
ASV_310	3.64E-05	Depleted	0.006	0.547	Bacteria	Firmicutes	Bacilli	Lactobacillales	Streptococcaceae	Streptococcus	Unassigned
ASV_97	4.63E-05	Enriched	0.244	0.027	Bacteria	Firmicutes	Negativicutes	Selenomonadales	Veillonellaceae	Veillonella	V.dispar
ASV_159	0.00011579	Depleted	0	0.446	Bacteria	Proteobacteria	Betaproteobacteria	Burkholderiales	Burkholderiales_incertae_sedis	Aquabacterium	A.commune
ASV_80	0.00011614	Depleted	0	0.862	Bacteria	Proteobacteria	Alphaproteobacteria	Rhodospirillales	Rhodospirillaceae	Elstera	E.litoralis
ASV_113	0.00025664	Enriched	0.278	0.097	Bacteria	Firmicutes	Clostridia	Clostridiales	Lachnospiraceae	Clostridium_XlVa	Unassigned
ASV_187	0.00030074	Enriched	1.866	0.146	Bacteria	Bacteroidetes	Bacteroidia	Bacteroidales	Bacteroidaceae	Bacteroides	B.fragilis
ASV_260	0.00049501	Depleted	0.005	0.58	Bacteria	Bacteroidetes	Bacteroidia	Bacteroidales	Prevotellaceae	Prevotella	P.copri
ASV_29	0.00062518	Enriched	1.12	0.364	Bacteria	Firmicutes	Clostridia	Clostridiales	Lachnospiraceae	Lachnospiracea_incertae_sedis	R.gnavus
ASV_314	0.0011407	Enriched	0.308	0.005	Bacteria	Bacteroidetes	Bacteroidia	Bacteroidales	Bacteroidaceae	Bacteroides	B.fragilis
ASV_111	0.00175788	Enriched	0.178	0.046	Bacteria	Firmicutes	Clostridia	Clostridiales	Lachnospiraceae	Ruminococcus2	R.torques
ASV_42	0.00250855	Depleted	0.177	0.987	Bacteria	Actinobacteria	Actinobacteria	Bifidobacteriales	Bifidobacteriaceae	Bifidobacterium	Unassigned
ASV_60	0.00304672	Enriched	0.479	0	Bacteria	Firmicutes	Clostridia	Clostridiales	Lachnospiraceae	Unassigned	Unassigned
ASV_8	0.00439836	Enriched	3.243	0.917	Bacteria	Proteobacteria	Gammaproteobacteria	Enterobacteriales	Enterobacteriaceae	Escherichia/Shigella	E.coli
ASV_137	0.00462641	Depleted	0.016	0.439	Bacteria	Proteobacteria	Alphaproteobacteria	Sphingomonadales	Sphingomonadaceae	Blastomonas	Unassigned
ASV_222	0.00732624	Enriched	0.191	0	Bacteria	Bacteroidetes	Bacteroidia	Bacteroidales	Porphyromonadaceae	Porphyromonas	P.bennonis
ASV_122	0.01181947	Enriched	0.407	0.001	Bacteria	Firmicutes	Clostridia	Clostridiales	Clostridiales_Incertae_Sedis_XI	Ezakiella	E.peruensis
ASV_135	0.01216059	Enriched	0.425	0.021	Bacteria	Bacteroidetes	Bacteroidia	Bacteroidales	Prevotellaceae	Prevotella	P.copri
ASV_92	0.0127408	Enriched	0.26	0.004	Bacteria	Proteobacteria	Betaproteobacteria	Burkholderiales	Sutterellaceae	Sutterella	S.stercoricanis
ASV_198	0.01355082	Enriched	0.109	0	Bacteria	Firmicutes	Negativicutes	Selenomonadales	Veillonellaceae	Megamonas	M.funiformis
ASV_119	0.01367849	Enriched	0.603	0.003	Bacteria	Firmicutes	Negativicutes	Selenomonadales	Veillonellaceae	Megamonas	M.funiformis
ASV_18	0.01648453	Enriched	3.312	0.37	Bacteria	Bacteroidetes	Bacteroidia	Bacteroidales	Prevotellaceae	Prevotella	P.copri
ASV_59	0.01668729	Enriched	0.641	0.024	Bacteria	Bacteroidetes	Bacteroidia	Bacteroidales	Prevotellaceae	Prevotella	P.copri
ASV_86	0.02369294	Enriched	0.368	0.026	Bacteria	Bacteroidetes	Bacteroidia	Bacteroidales	Prevotellaceae	Prevotella	P.copri
ASV_71	0.02696062	Enriched	0.307	0.098	Bacteria	Bacteroidetes	Bacteroidia	Bacteroidales	Rikenellaceae	Alistipes	A.onderdonkii

ASV_76	0.02768197	Enriched	0.331	0.04	Bacteria	Proteobacteria	Betaproteobacteria	Burkholderiales	Sutterellaceae	Sutterella	S.wadsworthensis
ASV_93	0.02872493	Enriched	0.589	0.001	Bacteria	Bacteroidetes	Bacteroidia	Bacteroidales	Porphyromonadaceae	Porphyromonas	P.bennonis
ASV_169	0.02895861	Enriched	0.211	0.078	Bacteria	Firmicutes	Clostridia	Clostridiales	Lachnospiraceae	Anaerostipes	A.hadrus
ASV_46	0.02967361	Enriched	0.48	0.307	Bacteria	Bacteroidetes	Bacteroidia	Bacteroidales	Bacteroidaceae	Bacteroides	Unassigned
ASV_48	0.03165644	Depleted	0.067	0.45	Bacteria	Bacteroidetes	Bacteroidia	Bacteroidales	Prevotellaceae	Paraprevotella	P.clara
ASV_73	0.0344558	Enriched	0.647	0.042	Bacteria	Bacteroidetes	Bacteroidia	Bacteroidales	Prevotellaceae	Prevotella	P.copri
ASV_95	0.03930653	Depleted	0.053	0.19	Bacteria	Bacteroidetes	Bacteroidia	Bacteroidales	Prevotellaceae	Paraprevotella	P.clara
ASV_140	0.0402965	Enriched	0.225	0.014	Bacteria	Bacteroidetes	Bacteroidia	Bacteroidales	Prevotellaceae	Prevotella	P.copri
ASV_267	0.04076079	Enriched	0.373	0.216	Bacteria	Bacteroidetes	Bacteroidia	Bacteroidales	Porphyromonadaceae	Parabacteroides	P.distasonis
ASV_9	0.04277035	Depleted	1.928	3.527	Bacteria	Firmicutes	Clostridia	Clostridiales	Ruminococcaceae	Faecalibacterium	F.prausnitzii
ASV_58	0.04458168	Enriched	0.348	0.186	Bacteria	Actinobacteria	Actinobacteria	Bifidobacteriales	Bifidobacteriaceae	Bifidobacterium	Unassigned

Table S2 Differential ASVs depiction between the AP and PR groups.

ID	P-Value	level	MeanPP	MeanPR	Kingdom	Phylum	Class	Order	Family	Genus	Species
ASV_198	1.03E-05	Depleted	0	0.365	Bacteria	Firmicutes	Negativicutes	Selenomonadales	Veillonellaceae	Megamonas	M.funiformis
ASV_178	2.25E-05	Depleted	0	1.765	Bacteria	Bacteroidetes	Bacteroidia	Bacteroidales	Bacteroidaceae	Bacteroides	B.graminisolvens
ASV_289	0.00010262	Depleted	0.008	0.453	Bacteria	Firmicutes	Clostridia	Clostridiales	Lachnospiraceae	Blautia	Unassigned
ASV_113	0.0002445	Depleted	0.097	0.485	Bacteria	Firmicutes	Clostridia	Clostridiales	Lachnospiraceae	Clostridium_XlVa	Unassigned
ASV_48	0.00026873	Enriched	0.45	0	Bacteria	Bacteroidetes	Bacteroidia	Bacteroidales	Prevotellaceae	Paraprevotella	P.clara
ASV_97	0.00083242	Depleted	0.027	0.223	Bacteria	Firmicutes	Negativicutes	Selenomonadales	Veillonellaceae	Veillonella	V.dispar
ASV_111	0.00378288	Depleted	0.046	0.172	Bacteria	Firmicutes	Clostridia	Clostridiales	Lachnospiraceae	Ruminococcus2	R.torques
ASV_59	0.00379387	Depleted	0.024	1.306	Bacteria	Bacteroidetes	Bacteroidia	Bacteroidales	Prevotellaceae	Prevotella	P.copri
ASV_36	0.00387991	Enriched	0.521	0.048	Bacteria	Bacteroidetes	Bacteroidia	Bacteroidales	Bacteroidaceae	Bacteroides	B.intestinalis
ASV_86	0.00507077	Depleted	0.026	1.986	Bacteria	Bacteroidetes	Bacteroidia	Bacteroidales	Prevotellaceae	Prevotella	P.copri
ASV_137	0.0059212	Enriched	0.439	0	Bacteria	Proteobacteria	Alphaproteobacteria	Sphingomonadales	Sphingomonadaceae	Blastomonas	Unassigned
ASV_68	0.00611322	Enriched	0.392	0	Bacteria	Firmicutes	Clostridia	Clostridiales	Ruminococcaceae	Faecalibacterium	F.prausnitzii
ASV_3	0.00713072	Enriched	2.253	0.208	Bacteria	Bacteroidetes	Bacteroidia	Bacteroidales	Bacteroidaceae	Bacteroides	Unassigned
ASV_114	0.00858757	Enriched	0.604	0	Bacteria	Proteobacteria	Alphaproteobacteria	Caulobacterales	Caulobacteraceae	Caulobacter	C.segnis
ASV_80	0.00931213	Enriched	0.862	0	Bacteria	Proteobacteria	Alphaproteobacteria	Rhodospirillales	Rhodospirillaceae	Elstera	E.litoralis
ASV_120	0.00943251	Depleted	0.019	0.68	Bacteria	Firmicutes	Clostridia	Clostridiales	Lachnospiraceae	Lachnospiracea_incertae_sedis	E.ruminantium
ASV_159	0.00983776	Enriched	0.446	0	Bacteria	Proteobacteria	Betaproteobacteria	Burkholderiales	Burkholderiales_incertae_sedis	Aquabacterium	A.commune
ASV_46	0.01025564	Enriched	0.307	0.072	Bacteria	Bacteroidetes	Bacteroidia	Bacteroidales	Bacteroidaceae	Bacteroides	Unassigned
ASV_66	0.01595594	Enriched	0.394	0.053	Bacteria	Bacteroidetes	Bacteroidia	Bacteroidales	Bacteroidaceae	Bacteroides	B.xylanisolvens
ASV_133	0.02135519	Depleted	0.086	0.774	Bacteria	Actinobacteria	Actinobacteria	Coriobacteriales	Coriobacteriaceae	Collinsella	C.aerofaciens
ASV_1	0.02331704	Enriched	6.036	3.248	Bacteria	Bacteroidetes	Bacteroidia	Bacteroidales	Bacteroidaceae	Bacteroides	B.dorei
ASV_74	0.02449485	Enriched	0.3	0.011	Bacteria	Proteobacteria	Betaproteobacteria	Burkholderiales	Sutterellaceae	Parasutterella	P.excrementihominis
ASV_174	0.02905127	Enriched	0.142	0.022	Bacteria	Firmicutes	Clostridia	Clostridiales	Ruminococcaceae	Faecalibacterium	F.prausnitzii
ASV_37	0.02913628	Enriched	0.289	0.012	Bacteria	Bacteroidetes	Bacteroidia	Bacteroidales	Bacteroidaceae	Bacteroides	Unassigned
ASV_189	0.02976519	Depleted	0.091	1.014	Bacteria	Fusobacteria	Fusobacteriia	Fusobacteriales	Fusobacteriaceae	Fusobacterium	Unassigned
ASV_12	0.03000444	Enriched	2.504	0.513	Bacteria	Firmicutes	Clostridia	Clostridiales	Ruminococcaceae	Faecalibacterium	F.prausnitzii

ASV_112	0.03237937	Depleted	0.011	0.699	Bacteria	Firmicutes	Negativicutes	Selenomonadales	Veillonellaceae	Megamonas	M.funiformis
ASV_20	0.03407427	Depleted	0.501	2.884	Bacteria	Bacteroidetes	Bacteroidia	Bacteroidales	Prevotellaceae	Prevotella	P.copri
ASV_10	0.03440743	Enriched	1.511	0.041	Bacteria	Bacteroidetes	Bacteroidia	Bacteroidales	Bacteroidaceae	Bacteroides	B.coprocola
ASV_73	0.03682104	Depleted	0.042	0.612	Bacteria	Bacteroidetes	Bacteroidia	Bacteroidales	Prevotellaceae	Prevotella	P.copri
ASV_18	0.04004046	Depleted	0.37	2.205	Bacteria	Bacteroidetes	Bacteroidia	Bacteroidales	Prevotellaceae	Prevotella	P.copri
ASV_126	0.04175634	Enriched	0.351	0.08	Bacteria	Bacteroidetes	Bacteroidia	Bacteroidales	Bacteroidaceae	Bacteroides	B.caccae
ASV_38	0.04327468	Enriched	0.388	0.025	Bacteria	Bacteroidetes	Bacteroidia	Bacteroidales	Bacteroidaceae	Bacteroides	B.massiliensis
ASV_216	0.0437908	Enriched	0.082	0	Bacteria	Firmicutes	Negativicutes	Selenomonadales	Veillonellaceae	Megamonas	Unassigned
ASV_6	0.04410208	Enriched	1.686	0.419	Bacteria	Bacteroidetes	Bacteroidia	Bacteroidales	Bacteroidaceae	Bacteroides	B.dorei

Table S3 Differential ASVs depiction between the AP and HCs groups.

ID	PValue	level	MeanHCs	MeanPR	Kingdom	Phylum	Class	Order	Family	Genus	Species
ASV_178	4.80E-06	Depleted	0	1.765	Bacteria	Bacteroidetes	Bacteroidia	Bacteroidales	Bacteroidaceae	Bacteroides	B.graminisolvens
ASV_48	0.00153242	Enriched	0.067	0	Bacteria	Bacteroidetes	Bacteroidia	Bacteroidales	Prevotellaceae	Paraprevotella	P.clara
ASV_46	0.00256004	Enriched	0.48	0.072	Bacteria	Bacteroidetes	Bacteroidia	Bacteroidales	Bacteroidaceae	Bacteroides	Unassigned
ASV_187	0.00306937	Enriched	1.866	0.065	Bacteria	Bacteroidetes	Bacteroidia	Bacteroidales	Bacteroidaceae	Bacteroides	B.fragilis
ASV_92	0.00394034	Enriched	0.26	0.001	Bacteria	Proteobacteria	Betaproteobacteria	Burkholderiales	Sutterellaceae	Sutterella	S.stercoricanis
ASV_3	0.00414757	Enriched	1.817	0.208	Bacteria	Bacteroidetes	Bacteroidia	Bacteroidales	Bacteroidaceae	Bacteroides	Unassigned
ASV_37	0.00539039	Enriched	0.286	0.012	Bacteria	Bacteroidetes	Bacteroidia	Bacteroidales	Bacteroidaceae	Bacteroides	Unassigned
ASV_51	0.00761636	Enriched	0.233	0.05	Bacteria	Proteobacteria	Gammaproteobacteria	Enterobacteriales	Enterobacteriaceae	Klebsiella	Unassigned
ASV_60	0.00809107	Enriched	0.479	0	Bacteria	Firmicutes	Clostridia	Clostridiales	Lachnospiraceae	Unassigned	Unassigned
ASV_10	0.00924383	Enriched	1.51	0.041	Bacteria	Bacteroidetes	Bacteroidia	Bacteroidales	Bacteroidaceae	Bacteroides	B.coprocola
ASV_36	0.00943903	Enriched	0.3	0.048	Bacteria	Proteobacteria	Betaproteobacteria	Burkholderiales	Sutterellaceae	Parasutterella	P.excrementihominis
ASV_65	0.00993268	Enriched	0.259	0.059	Bacteria	Proteobacteria	Gammaproteobacteria	Enterobacteriales	Enterobacteriaceae	Unassigned	Unassigned
ASV_174	0.01055764	Enriched	0.145	0.022	Bacteria	Firmicutes	Clostridia	Clostridiales	Ruminococcaceae	Faecalibacterium	F.prausnitzii
ASV_29	0.01253578	Enriched	1.12	0.816	Bacteria	Firmicutes	Clostridia	Clostridiales	Lachnospiraceae	Lachnospiracea_incertae_sedis	R.gnavus
ASV_34	0.01498544	Enriched	0.229	0.115	Bacteria	Proteobacteria	Gammaproteobacteria	Enterobacteriales	Enterobacteriaceae	Klebsiella	Unassigned
ASV_32	0.01666209	Enriched	0.677	0.02	Bacteria	Verrucomicrobia	Verrucomicrobiae	Verrucomicrobiales	Verrucomicrobiaceae	Akkermansia	A.muciniphila
ASV_97	0.02020691	Enriched	0.244	0.223	Bacteria	Firmicutes	Negativicutes	Selenomonadales	Veillonellaceae	Veillonella	V.dispar
ASV_137	0.02050938	Enriched	0.016	0	Bacteria	Proteobacteria	Alphaproteobacteria	Sphingomonadales	Sphingomonadaceae	Blastomonas	Unassigned
